# A method of rapid and cost-effective screening for small mutations in patients with Peutz-Jeghers Syndrome

**DOI:** 10.1186/1897-4287-10-S4-A11

**Published:** 2012-12-10

**Authors:** Paweł Boruń, Andrzej Plawski, Anna Bartkowiak, Wojciech Cichy

**Affiliations:** 1 Institute of Human Genetics Polish Academy of Sciences, Poznan, Poland; 2 Department of Pediatrics University of the Medical Sciences, Poznan, Poland

## 

Peutz-Jeghers syndrome (PJS, MIM # 175200) is a rare, hereditary predisposition characterized by the occurrence of hamartomatous polyps in the gastrointestinal tract, mucocutaneous pigmentation, and increased risk of cancer in multiple internal organs. The incidence of the syndrome, depending on the studied population, has estimated range from 1:25,000 even up to 1:300,000 births. PJS is an autosomal dominant disease and in most of the cases is caused by mutations in the STK11 gene (MIM # 602216) located on a small arm of chromosome 19 at position 19p3.3.

The majority of causative DNA changes identified in patients with PJS are small mutations, therefore, developing a rapid and cost-effective method of their detection is a key aspect in the advancement of genetic diagnostics of patients suffering from PJS. In our study we developed a methodology of detection of small mutations in entire coding sequence of the STK11 gene based on High Resolution Melting analysis (HRM).

In our group of 41 families with PJS small mutations of the STK11 gene were detected in 22 families (54%). In the remaining cases, where large rearrangements were not detected using MLPA, all of the coding exons were sequenced. However, this did not allow detection of any additional mutations. Therefore, the developed methodology of searching for small mutations using HRM allowed to detect all the STK11 gene sequence changes occurring in our group of patients. The study was financed by the Ministry of Education and Science, Poland, grant number N402 481537.

